# Interaction of KLF6 and Sp1 regulates basigin-2 expression mediated proliferation, invasion and metastasis in hepatocellular carcinoma

**DOI:** 10.18632/oncotarget.8564

**Published:** 2016-04-04

**Authors:** Ling-Min Kong, Li Yao, Ning Lu, Ya-Lu Dong, Jing Zhang, Yong-Qiang Wang, Lili Liu, He-Long Zhang, Jian-Guo Huang, Cheng-Gong Liao

**Affiliations:** ^1^ Department of Cell Biology, National Translational Science Center for Molecular Medicine, Fourth Military Medical University, Xi'an, 710032, P. R. China; ^2^ Department of Pathology, Tangdu Hospital, Fourth Military Medical University, Xi'an, 710038, P. R. China; ^3^ Department of Oncology, Urumqi General Hospital of Lanzhou Military Command of PLA, Urumqi, 830000, P. R. China; ^4^ Department of Oncology, Tangdu Hospital, Fourth Military Medical University, Xi'an, 710038, P. R. China; ^5^ Cancer Institute, Fourth Military Medical University, Xi'an, 710038, P. R. China

**Keywords:** KLF6, Sp1, basigin-2, hepatocellular carcinoma, metastasis

## Abstract

Accumulating evidence suggests that the tumor suppressor gene Krüppel-like factor 6 (KLF6) plays important roles in both development and progression of cancer. However, the role of KLF6 in hepatocellular carcinoma (HCC) remains unclear. Cancer-related molecule basigin-2 plays an important role in HCC progression and metastasis. Sp1, one of Sp/KLFs family members, regulates basigin-2 expression in HCC. The involvement of KLFs in basigin-2 regulation and HCC progression and metastasis has not been investigated. We first measured KLF6 expression levels in 50 pairs of HCC and adjacent normal tissues (ANTs) by immunohistochemistry. Specifically, low KLF6 expression but high Sp1 and basigin-2 expression were found in HCC tissues. By contrast, the ANTs showed high KLF6 expression but low Sp1 and basigin-2 expression. Kaplan–Meier analysis showed that higher expression of KLF6 was associated with better overall survival. The survival rate of KLF6-negative patients was lower than that of KLF6-positive patients (*P* = 0.015). We also found that KLF6 binds to the basigin-2 and Sp1 promoters and decreases their expression. Thus, we identified a microcircuitry mechanism in which KLF6 can repress basigin-2 expression directly by binding to its promoter or indirectly by inhibiting the expression of the transcription factor Sp1 to block gene expression. Additionally, overexpression of KLF6 suppressed the invasion, metastasis and proliferation of HCC cells *in vitro* and *in vivo* by targeting basigin-2. Our study provides new evidence that interaction of KLF6 and Sp1 regulates basigin-2 expression in HCC and that KLF6 represses the invasive and metastatic capacities of HCC through basigin-2.

## INTRODUCTION

Hepatocellular carcinoma (HCC) is one of the most common cancers in the world, and is also the third most common cause of cancer-related death in adults [1–3]. Although new therapeutic strategies have significantly improved survival for tumors detected at early stages, the majority of patients are still diagnosed at an advanced stage and their prognosis remains poor [4]. Invasion and metastasis of HCC are the main reason for its high mortality rate. Therefore, many studies have been conducted to investigate genes and gene products that drive the HCC metastatic process.

Krüppel-like factor 6 (KLF6) is a tumor suppressor gene that is functionally inactivated through a range of mechanisms in several types of cancer, including HCC [4–6]. KLF6 encodes a zinc finger protein that belongs to the family of Sp/KLF transcription factors that are composed of an N-terminal activation domain and 3 C2H2 zinc fingers. KLF6 is ubiquitously expressed in human tissues and regulates genes controlling cell cycle, apoptosis and differentiation [7, 8]. However, the function of KLF6 in HCC invasion and metastasis has not been investigated.

Basigin-2, also known as extracellular matrix metalloproteinase inducer (EMMPRIN), CD147 and HAb18G/CD147, is a 58-kDa transmembrane glycoprotein belonging to the immunoglobulin superfamily [9, 10]. Basigin-2 is highly expressed in many tumors, including breast cancer, lymphoma, oral squamous cell carcinoma, glioma, melanoma, lung, bladder, liver and kidney carcinomas [10–12]. It has been demonstrated that basigin-2 contributes significantly to tumor growth, metastasis and angiogenesis through stimulating the production of hyaluronan, multiple matrix metalloproteinases (MMPs) and vascular endothelial growth factor A (VEGF-A) [13]. Our previous studies have shown that the transcription factor Sp1 can bind to basigin-2 promoter motifs and regulate basigin-2 expression in lung and liver cancers [14, 15]. These motifs are also the cognate recognition sequences for KLF6. Regulation of target gene expression often occurs through the cooperativity of KLF6 and Sp1 through a direct physical interaction [16]. In this study, we determined whether KLF6 is involved in basigin-2 regulation and whether it participates in HCC progression and metastasis.

We first measured KLF6, Sp1 and basigin-2 expression levels in HCC tumor tissues compared with normal liver tissues, and HCC cell lines. We identified the role of KLF6 in Sp1 and basigin-2 expression regulation. Specifically, we identified a microcircuitry mechanism in which KLF6 can repress basigin-2 expression directly by binding to its promoter or indirectly by inhibiting the expression of transcription factor Sp1 to block gene expression. In addition, overexpression of KLF6 suppressed the invasion, metastasis and proliferation of HCC cells *in vitro* and *in vivo* by targeting basigin-2. Thus, our data suggest that KLF6 has an important role in HCC progression and that KLF6 is a potential target for HCC therapies.

## RESULTS

### Expression of KLF6 is down-regulated in HCC tissues and cell lines

**Figure 1:**
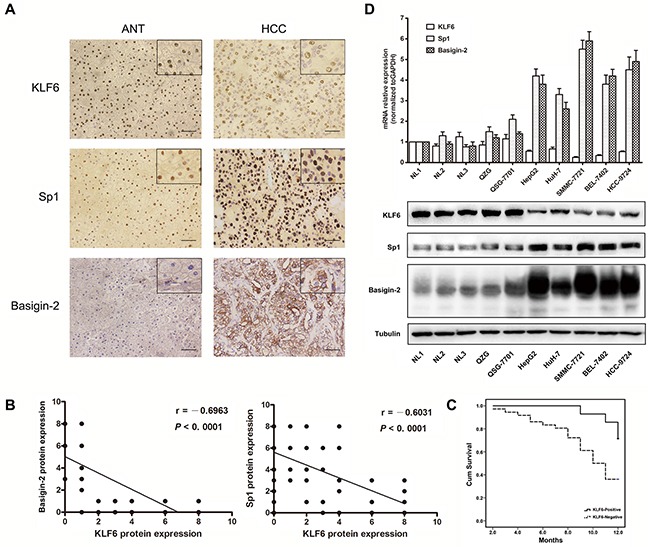
KLF6 is down-regulated in HCC tissues and cell lines **A.** IHC analysis of KLF6, Sp1 and basigin-2 protein expression in HCC and paired adjacent normal tissues. Pictures of representative areas are presented at different staining intensities (weak and strong) in ANT and tumor tissues. Scale bars, 50 μm. **B.** Spearman rank correlation analysis of KLF6 and Sp1 or basigin-2 protein expression levels in HCC and ANT tissues. **C.** Correlation of the overall survival rate of HCC patients with KLF6 expression pattern. Curves were estimated using the Kaplan–Meier method (*P* = 0.015). Continuous line KLF6 positive group; dotted line KLF6 negative group. **D.** The mRNA and protein expression of KLF6, Sp1, and basigin-2 were detected by real-time RT-PCR and western blotting.

The expression levels of KLF6 were first evaluated in fifty pairs of HCC and normal tissues by immunohistochemistry. As shown in Figure 1A, KLF6 was localized to the nuclei of hepatic cells. Twenty-six percent (13/50) of HCC specimens were positive for KLF6 expression, which was significantly lower than the 66% (33/50) in the adjacent tissues. The expression of basigin-2 and Sp1 were also detected. Basigin-2 was predominantly localized to the cytoplasm and membrane whereas the transcription factor Sp1 was localized to the nuclei of HCC cells. The positive expression rate of basigin-2 and Sp1 was 72% (36/50) and 68% (34/50) in HCC, respectively, and 18% (9/50) and 26% (13/50) in ANTs, respectively.

KLF6 protein expression was negatively correlated with basigin-2 and Sp1 (r = −0.6963, R squared = 0.4848, *P* < 0.0001 and r = − 0.6031, R squared = 0.3637, *P* < 0.0001, respectively) (Figure 1B). This correlation indicates that KLF6 may negatively regulate Sp1 and basigin-2 expression. Next, we examined whether down-regulation of KLF6 is correlated with HCC patient survival. Kaplan–Meier analysis showed that higher expression of KLF6 was correlated with higher overall survival (Figure 1C). The survival rate of KLF6-negative patients was lower than that of KLF6-positive patients, as determined using the log-rank test (*P* = 0.015). These results confirm that down-regulation of KLF6 is associated with advanced and aggressive tumor behaviors that are relevant to tumor metastasis and survival in HCC.

We further evaluated the expression levels of KLF6, Sp1 and basigin-2 in HCC cell lines by real-time RT-PCR and western blot analysis. The results showed that the mRNA and protein expression levels of basigin-2 and Sp1 were significantly increased in all tumorigenic HCC cell lines compared with non-tumorigenic HCC cell lines and normal liver tissues and cells. By contrast, KLF6 levels were lower in all HCC cell lines compared with normal liver tissues and cells (Figure 1D). These data suggest that the expression of KLF6 and Sp1/basigin-2 were mutually exclusive.

### KLF6 directly binds to the Sp1 and basigin-2 promoters

**Figure 2:**
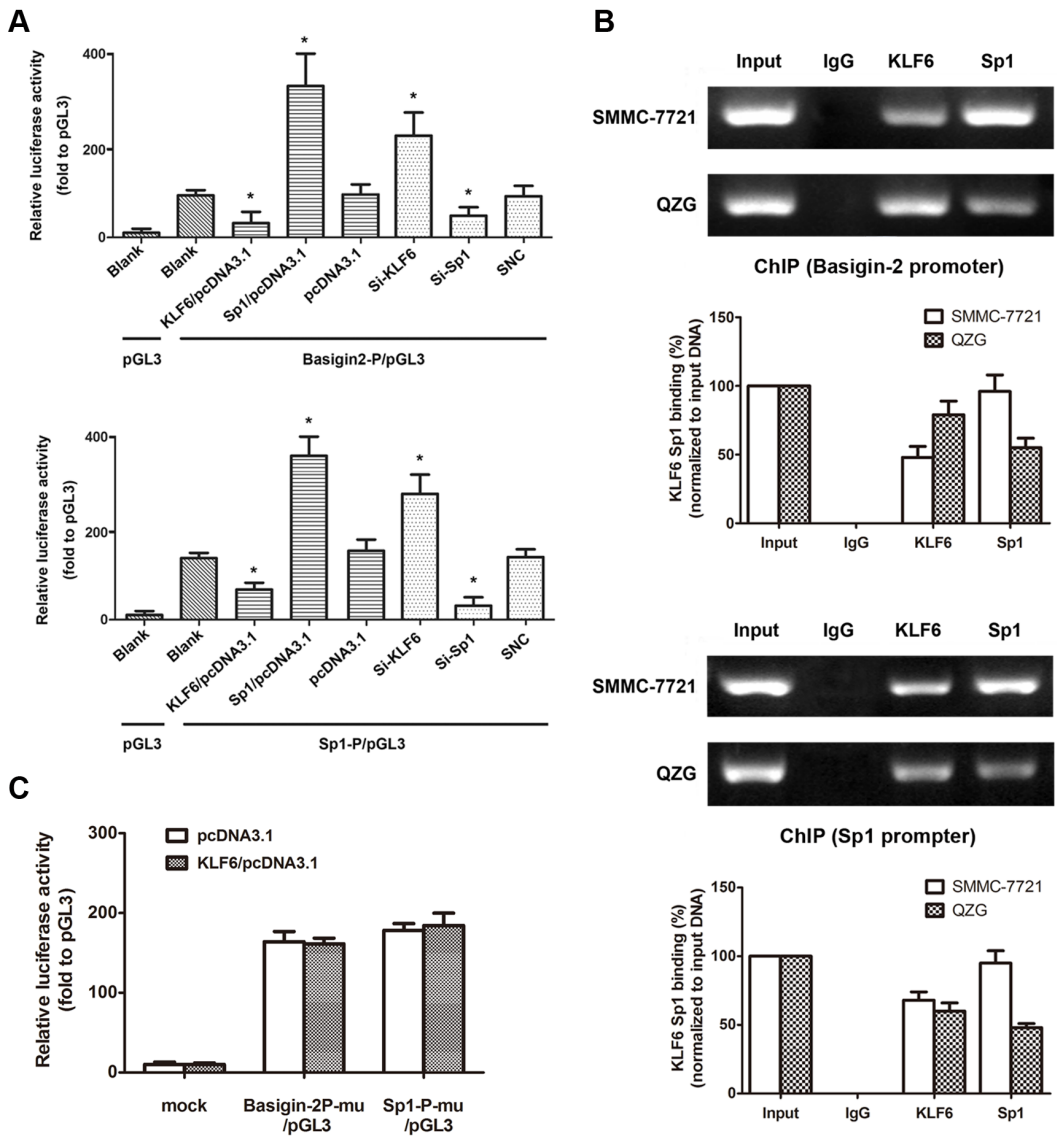
KLF6 directly binds to the Sp1 and basigin-2 promoter **A.** The dual-luciferase reporter assay was performed by co-transfecting reporter vectors inserted with the basigin-2 promoter (basigin-2P/pGL3) or the Sp1 minimal promoter (Sp1-P/pGL3) with overexpression vectors or knockdown siRNA of KLF6 and Sp1, respectively. *, *P* < 0.05, using Student's *t* test. **B.** The ChIP assay demonstrated endogenous KLF6 and Sp1 binds to the basigin-2 and Sp1 promoter. The histograms represent quantification of ChIP results. **C.** Reporter assay results in cells transfected with various Sp1 and basigin-2 promoter constructs with mutations in KLF6 binding elements. Mu, mutation type. Luciferase activity was expressed as relative to that of the pGL3 vector. *, *P* < 0.05, using Student's *t* test.

To determine the role of KLF6 in Sp1 and basigin-2 transcription, we cloned the human basigin-2 core promoter fragment (nucleotides −217 to +1) [15] and minimal Sp1 promoter into the pGL3 luciferase vector for a luciferase activity assay. The transcriptional activity of Sp1 and basigin-2 were reduced by KLF6 overexpression. Multiple siRNAs targeting KLF6 were designed and validated (Supplementary Figure S1). Silencing of KLF6 by pooled siRNA promoted the transcription activity of Sp1 and basigin-2 (Figure 2A). These results suggest that KLF6 participates in the regulation of Sp1 and basigin-2 transcriptional activity. We also confirmed that Sp1 could bind to its own promoter and upregulate its own transcriptional activity.

Furthermore, we performed *in vivo* ChIP assays to investigate whether KLF6 binds to basigin-2 and Sp1 promoter regions. We detected the protein levels that were pulled down in the ChIP assay by western blotting (Supplementary Figure S2). The ChIP assays revealed that endogenous KLF6 bound to basigin-2 and Sp1 promoters (Figure 2B). We also detected the binding of transcription factor Sp1 to the promoters of basigin-2 and Sp1. Interestingly, the binding of KLF6 in HCC cells was less than that in normal liver cells whereas the inverse was observed in case of Sp1.

To validate this notion, we mutated these binding sites individually and used them in a reporter assay. The results showed that the mutations in KLF6 binding sites in either the Sp1 promoter or basigin-2 core promoter significantly impaired the effect of KLF6 on Sp1 and basigin-2 transcription activation (Figure 2C), suggesting that KLF6 can bind to its special binding motifs on Sp1 and basigin-2 promoters to down-regulate their transcription. Additionally, we found that increased KLF6 clearly reduced the binding of Sp1 to the basigin-2 promoter. Increasing Sp1 got similar results (Supplementary Figure S3). The Sp/KLF family member Sp1 and KLF6 can bind to the basigin-2 and Sp1 promoter and regulate basigin-2 and Sp1 transcriptional activity.

### KLF6 negatively regulates Sp1 and basigin-2 expression

**Figure 3:**
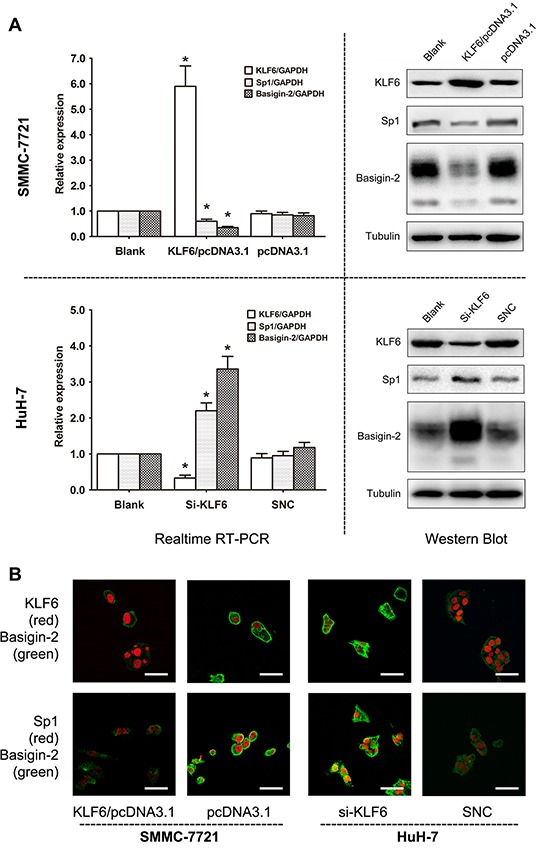
KLF6 negatively regulates Sp1 and basigin-2 expression **A.** KLF6, Sp1, and basigin-2 mRNA and protein expression in cells transfected with corresponding overexpression vector (upper) or siRNAs (lower). *, *P* < 0.05, using Student's *t* test. **B.** Expression of KLF6, Sp1, and basigin-2 in HCC cells transfected with overexpression vector or siRNA of KLF6, as detected by confocal laser scanning microscopy. Scale bars, 50 μm.

To further assess the biological roles of KLF6 in Sp1 and basigin-2 expression, we applied loss- and gain-of-function approaches. We showed down-regulation and upregulation of Sp1 and basigin-2 mRNA and protein expression in HCC cells upon ectopic expression and siRNA knockdown of KLF6, respectively (Figure 3A). The role of KLF6 in Sp1 and basigin-2 gene transcription were further elucidated by immunofluorescence. As shown in Figure 3B, we detected nuclear localization (red) of KLF6 and Sp1 protein whereas basigin-2 was localized in the cytoplasm and at cell membrane (green). The expression of Sp1 and basigin-2 altered with changes in KLF6 expression, which was consistent with the results presented in Figure 3A. Together, these results suggest that KLF6 serves as a transcription factor that inactivates Sp1 and basigin-2 transcription and down-regulates their expression.

### KLF6 decreases the invasive, metastatic and proliferative capacities of HCC cells *in vitro* via basigin-2 down-regulation

**Figure 4:**
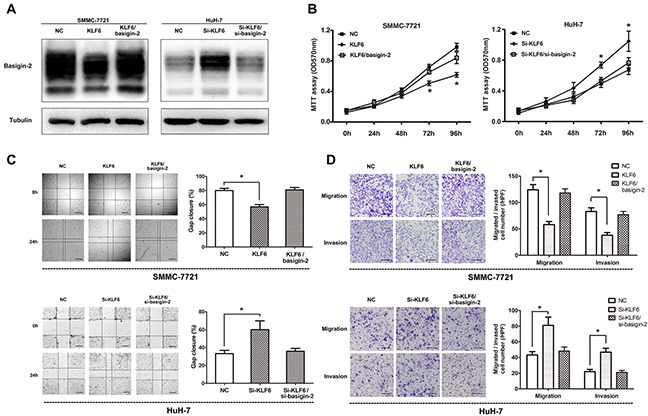
Overexpression of KLF6 inhibits the migration and invasion of HCC cell lines **A.** Western blot analysis of basigin-2 expression in SMMC-7721 and Huh-7 cells treated with KLF6 plasmid or siRNA. **B.** Cell proliferation of these cells transfected as in (A) was measured in the indicated time periods using MTT proliferation assays. *, *P* < 0.05, two-way repeated measures ANOVA followed by the Bonferroni test. **C.** Wound-healing assays of SMMC-7721 and Huh-7 cells transfected as in (A), compared with control, at 24 h after transfection. Scale bars, 500 μm. *, *P* < 0.05, by one-way ANOVA followed by the Dunnett test. **D.** The inhibitory effect of KLF6 toward the invasion and migration of SMMC-7721 and Huh-7 cells. Scale bars, 200 μm. *, *P* < 0.05, by one-way ANOVA followed by the Dunnett test. KLF6/basigin-2 means to co-transfect basigin-2/pcDNA3.1 with KLF6/pcDNA3.1, whereas Si-KLF6/si-basigin-2 means to co-transfect si-KLF6 with si-basigin-2.

Based on above results, we examined whether KLF6 can change the migration and invasion capacity of HCC cells. We transfected SMMC-7721 cells and Huh-7 cells with KLF6 expression vector or KLF6 siRNA and evaluated cell proliferation, invasion and migration. To confirm the role of KLF6 in HCC progression via its regulation on basigin-2 expression, we restored basigin-2 expression through transfecting a basigin-2 expression plasmid or siRNA to block KLF6 regulation. As expected, transfection of the KLF6 expression plasmid into SMMC-7721 cells resulted in decreased basigin-2 expression compared with the negative control (NC)-transfected cells. By contrast, si-KLF6 transfection increased basigin-2 expression in HuH-7 cells (Figure 4A).

To examine the role of KLF6 in the proliferation of HCC cells, we performed MTT cell proliferation assays in which KLF6 expression was altered in SMMC-7721 and Huh-7 cells. Our results showed that overexpression of KLF6 suppressed cell proliferation in SMMC-7721 cells and knock-down of KLF6 promoted cell proliferation in HuH-7 cells. However, restoring of basigin-2 expression blocked this effect (Figure 4B). Next, the wound-healing assay with SMMC-7721 and Huh-7 cells showed that overexpression of KLF6 presented a slower closing of scratch wound and knock-down of KLF6 resulted in a faster closing, compared with the negative controls (Figure 4C). Moreover, *in vitro* cell migration and invasion assays showed that overexpression of KLF6 inhibited migration and invasion ability of SMMC-7721 cells and knock-down of KLF6 boosted those of Huh-7 cells, compared with corresponding control (Figure 4D). At the same time, restoring of basigin-2 expression showed opposite effect on the invasive and metastatic capacities of HCC cells (Figures 4C and 4D). Our results indicate that KLF6 functions as a tumor suppressor and inhibits migration and invasion of HCC cells.

### KLF6 inhibits tumor growth, invasion and metastasis potential of HCC *in vivo*

**Figure 5:**
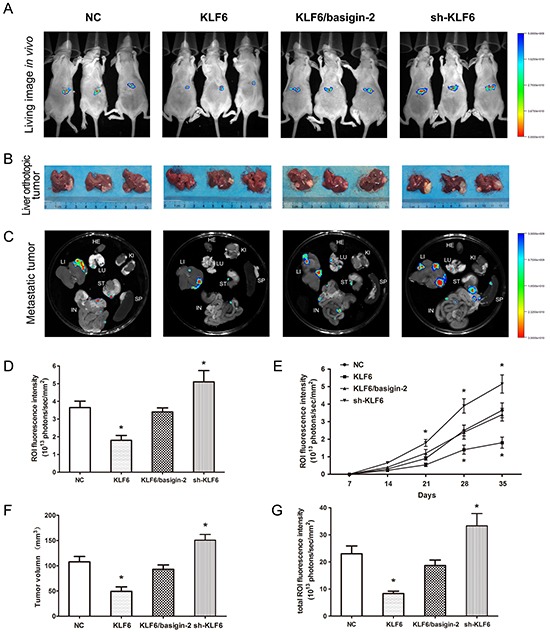
KLF6 inhibits HCC proliferation and invasion in a nude mouse model **A.** *In vivo* fluorescence images of the orthotopic HCC model in nude mice. The colored region represents the GFP fluorescence signal of HCC cells in nude mice. Right, signal intensity scale. Increasing red color indicates increasing signal strength, whereas increasing blue color indicates weaker signal strength. **B.** After the last imaging, the mice were sacrificed, and the livers were excised. The tumor size was measured in the resected liver. **C.** Selected organ images of mice on day 35 after inoculation. LU, lung; KI, kidney; LI, liver; SP, spleen; ST, stomach IN, intestine. **D.** Quantitative analysis of the fluorescence intensities in the four groups of (A). The ROI fluorescence intensity was recorded as photons/sec/mm^2^. *, *P* < 0.05, by one-way ANOVA followed by the Dunnett test. **E.** Effect of KLF6 on HCC cancer proliferation detected using *in vivo* imaging. *, *P* < 0.05, by two-way repeated measures ANOVA followed by the Bonferroni test. **F.** and **G.** Quantitative analysis of tumor volume and fluorescence intensities of (B) and (C). *, *P* < 0.05, by one-way ANOVA followed by the Dunnett test.

We next determined whether KLF6 overexpression could suppress tumor growth and metastasis *in vivo*. Using an orthotopic HCC model in nude mice, the negative control and KLF6/basigin-2 mice showed the apparent presence of GFP fluorescence emitted from primary tumor, whereas mice with KLF6 overexpression exhibited a lower GFP fluorescence signal at the observation endpoint. By contrast, knockdown of KLF6 expression significantly increased the GFP fluorescence signal (Figure 5A and 5D). The growth curve based on the data of living image repeated weekly revealed that the proliferation of KLF6 overexpressed tumors was slower than that of negative control. However, knockdown of KLF6 exhibited the fastest proliferation rate. Significant differences were observed between the fluorescence signals in KLF6 overexpressed mice and those in negative controls at days 28 and 35, and between the fluorescence signals in KLF6 knock-down mice and those in negative controls at days 21, 28 and 35 (*P* < 0.05; Figure 5E), suggesting that KLF6 exerted significant tumor growth suppression *in vivo*. All the mice were sacrificed after the last imaging, the tumors were excised and tumor volumes were measured. The KLF6 overexpressed mice had the smallest tumors, which were almost single tumors with no obvious live metastasis, whereas KLF6 knock-down mice presented the biggest tumors with multiple metastasis in liver (Figure 5B and 5F). We also compared tumor metastasis to important organs in these groups and were surprised to find that KLF6 overexpression resulted in obvious inhibition of distant metastasis to lung, omentum and mesenterium around stomach and small intestine (Figure 5C and 5G). Similarly, restoring of basigin-2 expression showed opposite effect on the growth, invasion and metastasis of HCC tumors compared with KLF overexpression mice (Figure 5). These findings suggest that KLF6 significantly inhibits proliferation, invasion and metastasis in HCC *in vivo* by down-regulating basigin-2.

### Interaction of KLF6 and Sp1 regulates basigin-2 expression

**Figure 6:**
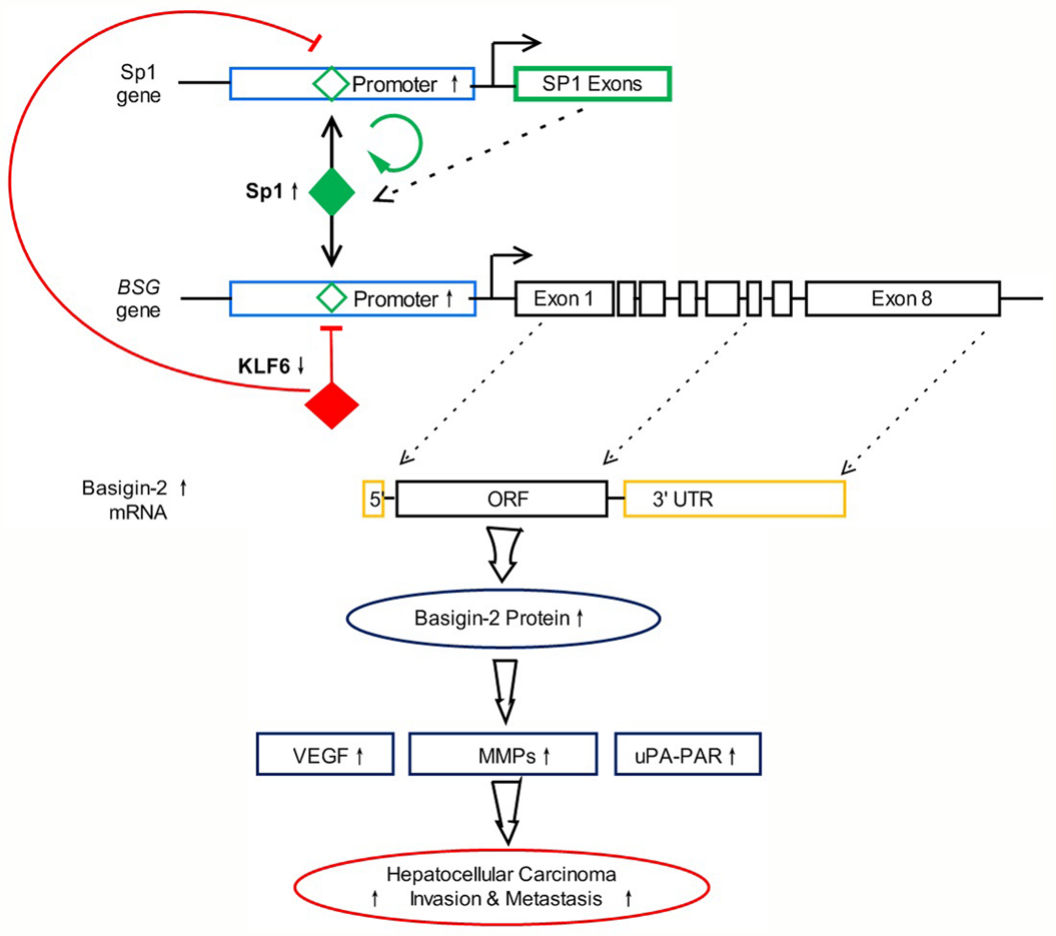
Summary diagram describing the interaction network of KLF6 and Sp1 regulate basigin-2 expression

Our previous studies validated that the transcription factor Sp1 could bind to the basigin-2 promoter motifs and regulate basigin-2 expression in HCC [14]. Our above results suggested that endogenous Sp1 is positively involved in basigin-2 promoter activity, whereas KLF6 functions in an opposite manner. KLF6 and Sp1 have antagonizing effects on the basigin-2 promoter activity. In addition, our results showed that Sp1 could activate its own transcription activity indicating that the Sp1 gene is autoregulated [17, 18]. Therefore, KLF6 down-regulates Sp1 expression through augmenting the negative regulation of KLF6 and attenuating positive autoregulation by Sp1. Altogether, we have identified a microcircuitry mechanism in which KLF6 can repress basigin-2 expression directly by binding to its promoter or indirectly by inhibiting the expression of the transcription factor Sp1 to block gene expression. The interaction of KLF6 and Sp1 regulates basigin-2 expression and is involved in processes such as cell proliferation, invasion and metastasis that are mediated by basigin-2 and its downstream genes in HCC. A summary diagram that outlines the above-described regulatory network is shown in Figure 6.

## DISCUSSION

In our study, we found that the expression of KLF6 was downregulated in HCC tissues and cell lines, whereas Sp1 and basigin-2 were upregulated. Kaplan–Meier analysis showed that higher expression of KLF6 was related to increased overall survival. The survival rate of KLF6-negative patients was lower than that of KLF6-positive patients. We found that KLF6 could directly bind to the Sp1 and basigin-2 promoters and inhibited their expression. Therefore, we identified a microcircuitry mechanism in which KLF6 could repress basigin-2 expression directly by binding to its promoter or indirectly by inhibiting the expression of transcription factor Sp1 to block gene expression. In addition, overexpression of KLF6 suppressed the invasion, metastasis and proliferation of HCC cells *in vitro* and *in vivo* by targeting basigin-2 both. Our study provides the first evidence that the interaction of KLF6 and Sp1 regulates basigin-2 expression in HCC and that KLF6 represses invasive and metastatic capacities through basigin-2 in HCC.

Invasion and metastasis, two of the most important hallmarks of malignant tumors, are the prominent fatal factors in human cancers [19]. Therefore, many studies have been conducted to investigate genes and gene products that drive the metastatic process. Previous studies have observed the loss of KLF4 staining in primary HCC, particularly the metastasis specimens. Reduced KLF4 expression was significantly correlated with advanced tumor biology and poor patient survival [20]. Because KLF6 and KLF4 bind to similar DNA sequences, we investigated the role of KLF6 in the HCC. KLF6 plays a crucial role in tumor suppression by modulating the expression of a broad range of genes governing biological functions associated with cell growth, differentiation, adhesion and endothelial motility [21]. Our work showed that KLF6 was more significantly down-regulated in HCC specimens than in the adjacent tissues and that this down-regulation correlated with the survival rate of HCC patients, indicating that KLF6 may serve as a new prognostic biomarker in HCC.

The inactivation of KLF6 by loss of heterozygosity (LOH) and/or mutation occurs in many types of tumors [5, 22]. Promoter hypermethylation has been reported to participate in the inactivation too [23]. Recently, a unique mechanism of KLF6 inactivation has been identified wherein alternatively spliced isoforms of KLF6 are generated that antagonize the tumor suppressive functions of the full-length, wtKLF6 protein [24, 25]. Three alternative splice variants of KLF6 termed SV1, SV2, and SV3 have been identified. An increased SV1/KLF6 mRNA ratio has been observed in HCC samples, which antagonizes wtKLF6 function [4]. The SV2 variant is down-regulated in HCC and displays anti-proliferative and pro-apoptotic functions [26]. Our work has validated the expression of all variants by variant-specific PCR. However, the expression levels of all alternative splice variants were fairly low in HCC cells and tissues. So we focused on the roles of wild type KLF6 protein in HCC.

Our previous work showed that basigin-2 was more strongly upregulated in HCC specimens than in the adjacent tissues and that this overexpression correlated with tumor metastasis and advanced histologic grades [11, 27, 28]. The transcription factor Sp1 can bind to the basigin-2 promoter motifs and regulate basigin-2 expression. Sp1 is usually recognized as a transcriptional activator of various genes involved in almost all cellular processes in mammalian cells [29]. Sp1 also participates in cancer development and progression [30, 31]. The current study provides a novel mechanism for the regulation of basigin-2 expression. Endogenous Sp1 is positively involved in basigin-2 promoter activity, whereas KLF6 has an opposite function. KLF6 and Sp1 have antagonizing effects on the basigin-2 promoter activity. Altogether, we have identified a microcircuitry mechanism in which KLF6 could repress basigin-2 expression directly by binding to its promoter or indirectly by inhibiting the expression of transcription factor Sp1 to block gene expression. Physiologically, the balance between Sp1 and KLF6 expression may play a critical role in the homeostasis of liver. However, during HCC development and progression, alteration of KLF6 expression that changes Sp1 expression may finally lead to aberrant basigin-2 expression. This mechanism may not be limited to HCC but rather a common mechanism in the progression of other cancers, because Sp1 overexpression and KLF6 down-regulation have also been reported in many cancers [32, 33].

Because of the central role of KLF6 in the mechanisms of basigin-2 regulation, we investigated its role in HCC progression. Gain-of-function assays were performed to assess the effects of KLF6 on HCC invasion and metastasis. The results showed that overexpression of KLF6 inhibited basigin-2 expression as well as cell proliferation, invasion and metastasis *in vitro*. Overexpression of KLF6 significantly suppressed tumor growth and metastasis in a mouse model of HCC metastasis, indicating the therapeutic potential of KLF6 in HCC metastasis. The identification of KLF6 as an important regulator of HCC cell migration and invasion emphasizes an essential role of this tumor suppressor gene in mediating HCC oncogenesis and tumor behavior [34].

In conclusion, KLF6 is down-regulated in HCC and inhibits cell migration and invasion of HCC cells *in vitro* and *in vivo*. Interaction of KLF6 and Sp1 plays an important role in basigin-2 transcription regulation. This newly identified KLF6/basigin-2 link provides a new, potential therapeutic target to treat HCC.

## MATERIALS AND METHODS

### Tissue specimens and immunohistochemical analysis

Fifty paired tissue specimens of HCC and matched adjacent normal tissues (ANTs) were collected from Tangdu Hospital of Fourth Military Medical University (Xi'an, China) from 2011 to 2012 and were histologically confirmed by staining with hematoxylin and eosin (H&E). Three fresh normal liver tissues (NT) were also collected as normal controls. All individuals provided written informed consent, and the study was approved by the hospital's Ethics Committee.

Immunohistochemistry was performed using Histostain-SP kits (Invitrogen, Carlsbad, CA, USA) according to the manufacturer's instructions. Antibodies were purchased from Santa Cruz Biotechnology (Santa Cruz, CA, USA). Immunopositivity was independently evaluated by two pathologists. Expression of protein was evaluated as described previously [15, 35].

### Cell lines and culture conditions

The following cell lines were used in this study: human normal liver cell QZG [36] and QSG-7701 [37]; human hepatocellular carcinoma cell lines: HepG2, Huh-7, SMMC-7721, BEL-7402 and HCC-9724 [38]. All cell lines were purchased from the Shanghai Institute for Biological Sciences (Shanghai, China). All cell lines were routinely cultured using standard protocols. Cell line authentication was assessed using short tandem repeat (STR) DNA profiling method every year in our laboratory and the latest verification was done in March 2013.

### Real-time quantitative RT-PCR

Real-time quantitative RT-PCR was performed as described previously [14]. Expression data were uniformly normalized to glyceraldehyde-3-phosphate dehydrogenase (GAPDH) as an internal control, and the relative expression levels were evaluated using the ΔΔCt method [12, 39]. Primers were used as described previously [12, 40, 41]. The oligonucleotide sequences of PCR primers are listed in Supplementary Table S1.

### Western blot analysis

Cell samples were lysed with RIPA buffer (Beyotime, China). Equal amounts (10 μg) of total protein were loaded, and then subsequently immunoblotted with the primary antibodies, including anti-basigin-2, Sp1, KLF6 and tubulin monoclonal antibodies (Santa Cruz, CA, USA). Proteins were detected using the Amersham enhanced chemiluminescence system (Pierce, Rockford, IL, USA) according to the manufacturer's instructions.

### Vector construction, siRNA, and luciferase reporter assay

The core promoter of the basigin-2 gene (−217 to +1, relative to the transcription start site of the basigin-2 gene) was constructed as previously described [15, 40]. The minimal promoter of Sp1 (−281 to -20, relative to the transcription start site of Sp1 gene) was amplified and cloned into the pGL3 plasmid as previously described [17]. To generate the site-directed mutants of KLF6 binding element of basigin-2 or Sp1 promoter, a QuickChange mutagenesis kit (Stratagene, La Jolla, CA, USA) was used as described previously [18, 40]. The coding sequences of Sp1 and KLF6 were amplified from the cDNA template of SMMC-7721 cells and cloned into pcDNA3.1 (Invitrogen, Carlsbad, CA, USA). Pooled multiple siRNAs targeting KLF6 (pooling si-KLF6-374, si-KLF6-554 and si-KLF6-682 in equal proportion), Sp1 and basigin-2 [42] were synthesized by Genepharma (Shanghai, China). All constructs were further confirmed by sequencing. All the oligonucleotide sequences of PCR primers and siRNA fragments are listed in Supplementary Table S1. Cell transfection and dual luciferase reporter assay were performed as described previously [40].

### Immunofluorescence

Cells were seeded in 4-well 35-mm dishes (Greiner Bio-One North America Inc., Monroe, NC, USA) at a density of 1,000 cells/well and grown for 48 h in culture medium. Then cells were fixed in 4% paraformaldehyde for 20 min and permeabilized in phosphate-buffered saline (PBS) supplemented with 0.5% Triton X-100. After blocking, cells were incubated with the indicated antibodies for 2 h. Cells were washed in PBS, incubated with their corresponding FITC-labeled secondary antibodies (Pierce) for 1 h at room temperature and stained with DAPI (Vector Labs, Burlingame, CA, USA). Finally, the cells were mounted using glycerol and observed using a Nikon A1 laser scanning confocal microscope (Japan).

### Chromatin immunoprecipitation (ChIP)

ChIP assays were performed using a EZ ChIP Assay Kit (Millipore Corporation, Billerica, MA, USA). DNA was quantified using RT-PCR. The antibodies used were: anti-KLF6, anti-Sp1 or IgG antibodies (Santa Cruz, CA, USA). The ChIP assay was performed as described previously [40]. Oligonucleotide sequences of PCR primers were listed in Supplementary Table S1.

### Cell proliferation assay

Cells were plated in sextuplicate in 96-well plates (2 × 10^3^ per well) in 100 μL complete medium and allowed to adhere overnight. 3-(4,5-dimethyl-2-thiazolyl)-2,5-diphenyl-2H-tetrazolium bromide (MTT) (20 μL at 5 mg/mL; Sigma, St. Louis, MO, USA) was added every 24 h and incubated for 4 h. The supernatant was discarded, the precipitate was dissolved in 200 μL dimethyl sulfoxide (DMSO), and plates were read with a microplate reader at 570 nm [43].

### *In vitro* invasion assay and migration assay

*In vitro* invasion assays were performed as previously described [12] with MilliCell chambers (Millipore). The migration assays were performed in the same way as the invasion assay, except that no Matrigel was used and the cell permeating time was 12 hours.

### Wound-healing assay

The wound-healing assay was used to evaluate tumor cell motility capacity. Briefly, 1 × 10^6^ cells were seeded in six-well plates, cultured overnight, and transfected with KLF6 or controls. When the culture reached nearly 90% confluency, the cell layer was scratched with a sterile plastic tip and then washed with culture medium twice and cultured again for up to 24 with serum-reduced medium containing 1% FBS. At different time points, photographic images of the plates were acquired under a microscope and the data were summarized based on sextuple assays for each experiment.

### Orthotopic HCC model, *in vivo* fluorescence imaging, and animal studies

Female BALB/c nu/nu mice at 4 to 6 weeks of age were provided by the Laboratory Animal Research Center of FMMU, and the animal study was reviewed and approved by the Animal Care and Use Committee. The SMMC-7721 cell stably expressing GFP was prepared previously and used as negative control (NC). Then, the cells were respectively stably transfected with KLF6/pcDNA3.1, KLF6/pcDNA3.1 rescued with basigin-2/pcDNA3.1 and KLF6 shRNA vector, and resuspended in 100 μl Matrigel and injected subcutaneously into the right flanks of nude mice [12]. When tumors reached a size of ∼1 cm^3^, the mice were sacrificed. The tumors were resected, cut into 1-mm^3^ sections under aseptic conditions, and then implanted under the liver capsules of the left hepatic lobes of nude mice. The health states and body weights of mice were observed every other day.

The animals were imaged weekly for 35 days using a Carestream MS FX Pro i*n vivo* imaging system (Carestream Health, Cheektowaga, NY, USA). For *in vivo* fluorescence imaging, mice were anesthetized with isoflurane, and a whole-body [image was acquired for 20 s with an excitation filter at 480 nm and an emission filter at 535 nm. Another image with an excitation filter at 430 nm was acquired for elimination of the nonspecific fluorescent background from skin and muscle. The region of interest (ROI) was drawn over the liver area and quantified using Carestream MI image analysis software. Fluorescence signals were normalized to photons per second per millimeter squared (photons/sec/mm^2^).

After the last imaging, mice were sacrificed, and livers were excised. Tumor volume was determined using direct measurement and calculated using the formula length × width^2^/2. Other organs (lung, stomach, spleen, kidney, and intestines) were examined for metastases.

### Statistical analysis

All statistical analyses were performed using the SPSS statistical software package (version 16.0, Chicago, IL). Each *in vitro* quantitative test was independently replicated, and all data are presented as mean ± SEM. One-way ANOVA or Student's *t* test was used to compare the expression levels, luciferase activities, migrated or invased cell numbers, fluorescence signals, tumor volumes, and grayscale values of immunohistochemistry staining among the different groups. Two-way repeated measures ANOVA was used to analyze the repeated measured data in proliferation assays. Follow-up time was limited to 1 year. Survival rates were compared by Kaplan–Meier test and log-rank test. The Spearman's rank correlation coefficient was used as a statistical measure of association. All the statistical tests were two sided, and *P* < 0.05 was considered with statistical significance.

## SUPPLEMENTARY FIGURES AND TABLES




