# Supplementary material for: Interaction of KLF6 and Sp1 regulates basigin-2 expression mediated proliferation, invasion and metastasis in hepatocellular carcinoma
Source: Oncotarget. 2016 Apr 4;7(19):27975–87. doi: 10.18632/oncotarget.8564 (PMC5053703; doi:10.18632/oncotarget.8564)
Supplement: Supplementary file 1 [file oncotarget-07-27975-s001.pdf]

SUPPLEMENTARY FIGURES AND TABLE

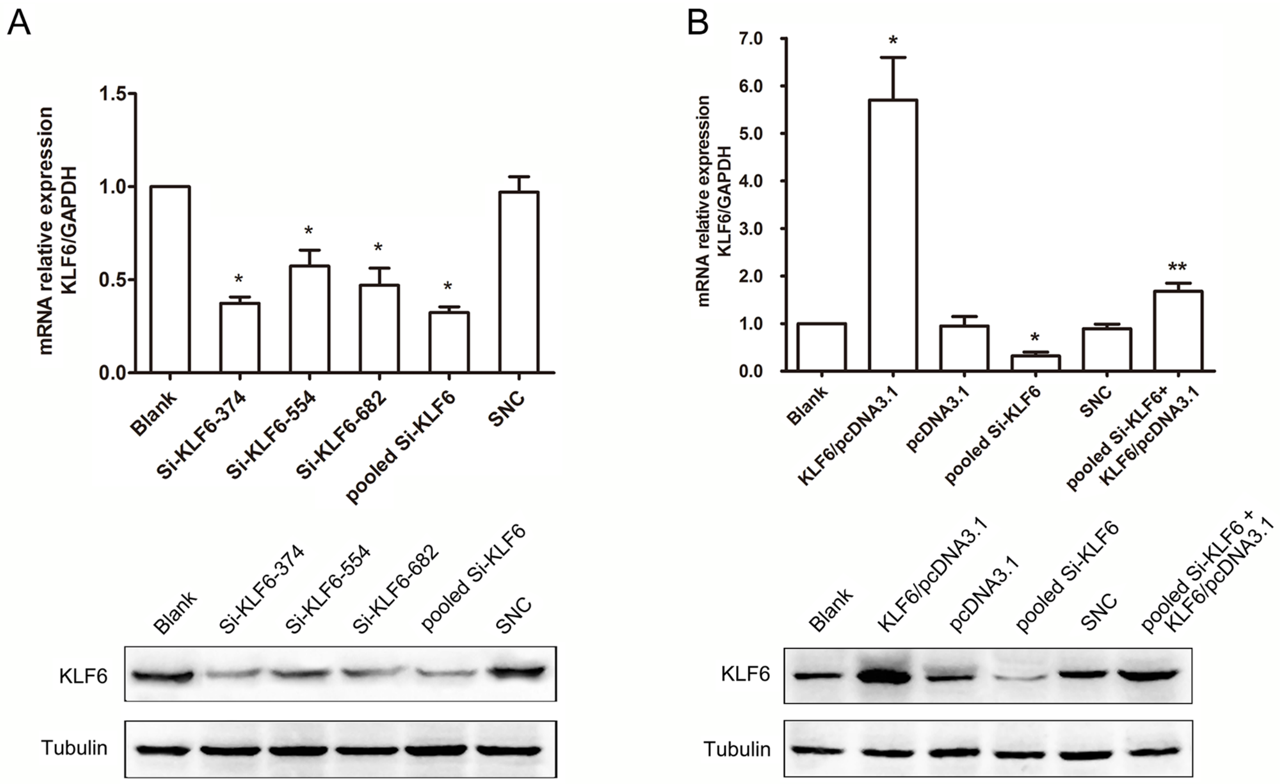

Supplementary Figure S1: The effect of KLF6 siRNA was detected and rescue experiments were conducted to exclude non-specificity. A. The KLF6 mRNA and protein expression levels in cells transfected with three single and pooled multiple siRNAs targeting KLF6 which were designed by Dharmacon siDESIGN Center tool in this work and named as si-KLF6-374, si-KLF6-554 and si-KLF6-682 according to the binding position on KLF6 mRNA (Accession number NM_001300) were detected by real-time quantitative RT-PCR and western blotting, respectively. *, $P < 0.05$, using Student's *t* test. **B.** Expression of KLF6 in HCC cells transfected with overexpression vector or pooled multiple siRNA of KLF6 were detected to rescue the RNAi effect by real-time quantitative RT-PCR and western blotting. *, $P < 0.05$, using Student's *t* test.

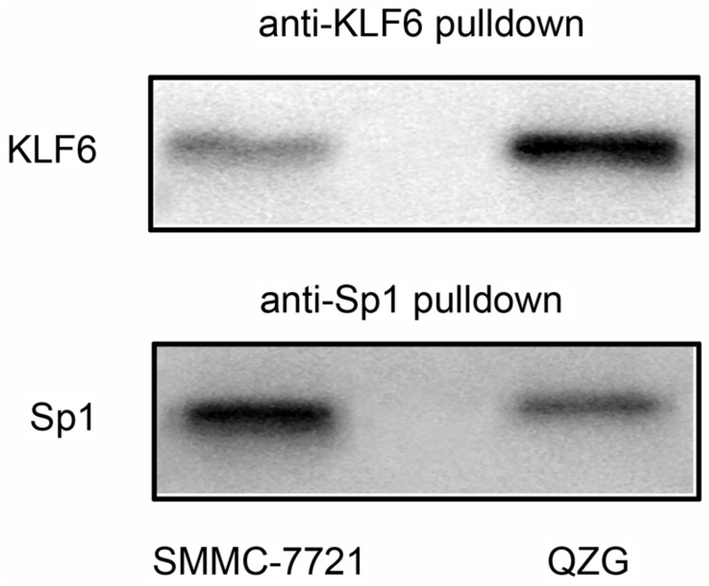

Supplementary Figure S2: The protein levels that were pulled down in ChIP were detected by western blotting.

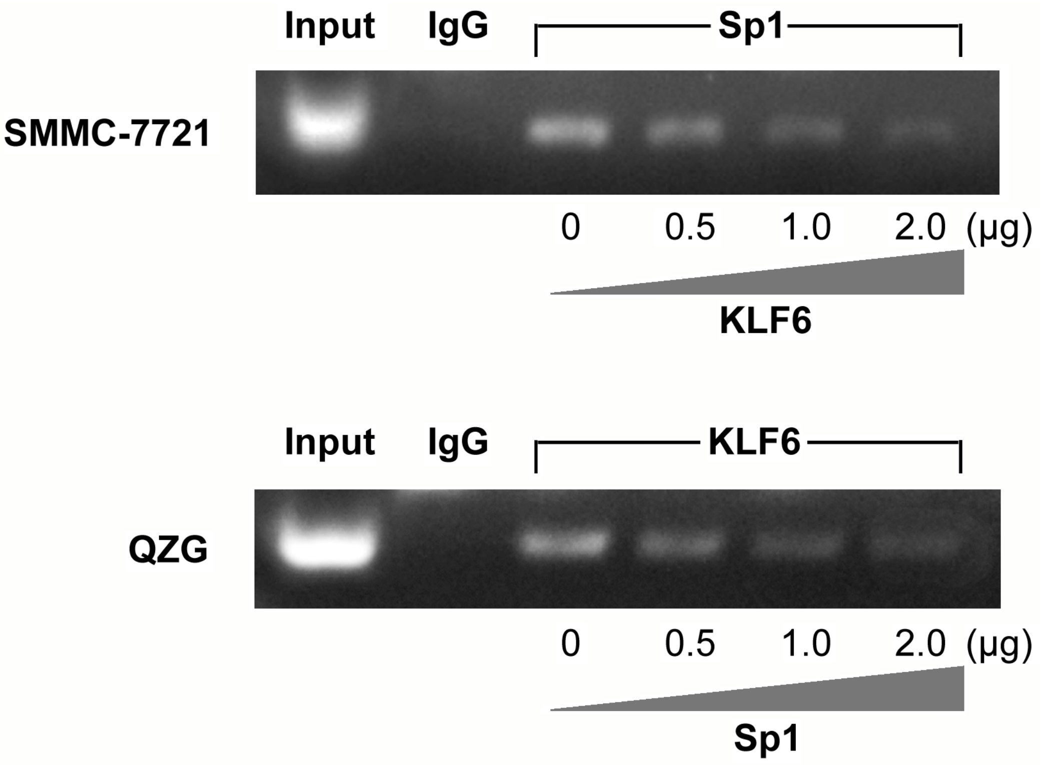

Supplementary Figure S3: The binding manner of KLF6 and Sp1 was detected by ChIP. Transfection with various differing doses of expression vector of KLF6 in SMMC-7721 cells or expression vector of Sp1 in QZG cells, respectively. The binding to the basigin-2 promoter were measured by ChIP.

Supplementary Table S1: Oligonucleotide sequences of PCR primers and siRNAs.

See Supplementary File 1
