# Supplementary material for: Interaction of KLF6 and Sp1 regulates basigin-2 expression mediated proliferation, invasion and metastasis in hepatocellular carcinoma
Source: Oncotarget. 2016 Apr 4;7(19):27975–87. doi: 10.18632/oncotarget.8564 (PMC5053703; doi:10.18632/oncotarget.8564)
Supplement: Supplementary file 2 [file oncotarget-07-27975-s002.doc]

**Supplementary table 1. Oligonucleotide sequences of PCR primers and siRNAs.**

| **Primers for real-time quantitative RT-PCR** | | |
| --- | --- | --- |
| Basigin-2 | 5’-TCGCGCTGCTGGGCACC-3’ | 5’-TGGCGCTGTCATTCAAGGA-3’ |
| KLF6 | 5’-CAAGGGAAATGGCGATGCCT-3’ | 5’-CTTTTCTCCTGTGTGCGTCC-3’ |
| Sp1 | 5’-AATTTGCCTGCCCTGAGTGC-3’ | 5’-TTGGACCCATGCTACCTTGC-3’ |
| GAPDH | 5’-AGCAATGCCTCCTGCACCACCAAC-3’ | 5’-CCGGAGGGGCCATCCACAGTCT-3’ |
| **Primers for expression plasmid construction** | | |
| Sp1 | 5’-ATCG*GGTACC***ATG**AGCGACCAAGATCACTCCAT-3’(*KpnI*) | 5’-ATCG*CTCGAG***TCA**GAAGCCATTGCCACTGATAT-3’(*Xho I*) |
| KLF6 | 5’-ATCG*GGTACC***ATG**GACGTGCTCCCCATGT-3’(*KpnI*) | 5’-ATCG*CTCGAG***TCA**GAGGTGCCTCTTCATGTG-3’(*Xho I*) |
| Basigin-2 | 5’-ATCG*GGTACC***ATG**GCGGCTGCGCTGTTCGT-3’(*KpnI*) | 5’-ATCG*CTCGAG***TCA**GGAAGAGTTCCTCTGGCG-3’(*Xho I*) |
| **Primers for luciferase plasmids construction** | | |
| Basigin-2P/pGL3 | 5’-ATCG*GCTAGC*CCGTTTCCTAGCAACGCCG-3’ (*Nhe I*) | 5’-ATCG*AAGCTT*GATTCCTATTCCTCGCCGGT-3’ (*Hind III*) |
| Sp1-P/pGL3 | 5’-ATCG*GCTAGC*GCAACTTAGTCTCACACGCCTTGG -3’ (*Nhe I*) | 5’-ATCG*AAGCTT*GCTCAAGGGGGTCCTGTCCGG-3’ (*Hind III*) |
| **Primers for ChIP assay promoter-specific PCR** | | |
| Basigin-2 promoter | 5’-ACATATGAGCTCGAAGCGCCGGAAG-3’ | 5’-TAATAGCGGCCGCGAGGTGAGAAC-3’ |
| Sp1 promoter | 5’-GGGCTTGTGGCGCGCTGCTC-3’ | 5’-GCTCAAGGGGGTCCTGTCCGG-3’ |
| **siRNA** | | |
| si-KLF6-374 | 5'-GCCUAGAGCUGGAACGUUAuu-3' | 5'-UAACGUUCCAGCUCUAGGCuu-3' |
| si-KLF6-554 | 5'-GUUACAACUUAGAGACCAAuu-3' | 5'-UUGGUCUCUAAGUUGUAACuu-3' |
| si-KLF6-682 | 5'-AAAUUGAGCUCCUCUGUCAuu-3' | 5'-UGACAGAGGAGCUCAAUUUuu-3' |
| si-Basigin-2-1 | 5’-GGUUCUUCGUGAGUUCCUCuu-3’ | 5’-GAGGAACUCACGAAGAACCuu-3’ |
| si-Basigin-2-2 | 5’-AGUCGUCAGAACACAUCAACGuu-3’ | 5’-CGUUGAUGUGUUCUGACGACUuu-3’ |
| si-Sp1-320 | 5’-AAUGAGAACAGCAACAACUuu-3’ | 5’-AGUUGUUGCUGUUCUCAUUuu-3’ |
| si-Sp1-590 | 5’-CCUGGAGUGAUGCCUAAUAuu-3’ | 5’-UAUUAGGCAUCACUCCAGGuu-3’ |
